# Knowledge and practice on prevention of diabetes mellitus among Diabetes mellitus family members, in suburban cities in Ethiopia

**DOI:** 10.1186/s13104-017-2871-7

**Published:** 2017-11-02

**Authors:** Mistire Wolde, Nega Berhe, Irma van Die, Girmay Medhin, Aster Tsegaye

**Affiliations:** 1 Aklilu Lemma Institute of Pathobiology, Addis Ababa University, Addis Ababa, Ethiopia; 2 Department of Medical Laboratory Sciences, College of Health Science, Addis Ababa University, Addis Ababa, Ethiopia; 3 Department of Molecular Cell Biology and Immunology, VU University Medical Center, Virje University, Amsterdam, The Netherlands

**Keywords:** Diabetes mellitus, DM family members, DM awareness, Ethiopia

## Abstract

**Background:**

Diabetes mellitus (DM) is one of the serious non communicable diseases worldwide. Presence of DM patient in a family may be considered as risk factor for other family members to acquire the disease, due to DM inheritance nature and/or similar life style pattern among family members. This paper assessed awareness of DM patients’ family members (DMPFMs) about DM occurrence and prevention. A cross sectional study was conducted in 2014 in two suburban cities of Ethiopia, namely Kemisse, and Kombolcha using an interviewer administered questionnaire among primary or secondary degree DMPFMs and controls. Based on eligibility criteria study participants were selected by health extension workers on house to house visit. Data were analyzed using SPSS version 20, and *P* value less than 0.05 considered as statistically significant.

**Results:**

Of the total 347 study participants, 45.5% (n = 158) had DMPFMs. Majority, 60.8% of DMPFMs and 73.0% of controls were males. Mean age of DMPFMs (30.06 years) was less than that of the controls (37.38 years). On living style, 51.9% DMPFMs, and 42.8% of controls were single. In both study groups, the majority of study participants attended grade 7–12. The likelihood of having good level of knowledge among DMPFMs were 2.94 times (AOR = 2.94 95% CI 1.87–4.86) higher compared to those who did not. Those attaining higher educational levels were 3.41 times (AOR = 3.41, 95% CI 1.31–8.91) more likely to have good level of knowledge, as compared to those who were unable to read and write. The likelihood of having good level of positive practice among DMPFMs were 3.38 times (AOR = 3.38% CI 2.05–5.58) higher as compared to controls. Participants who were living in Kombolcha were 2.33 times (AOR = 2.33 95% CI 1.31–4.12) more likely to have good level of practice, as compared to individuals from Kemisse.

**Conclusions:**

Diabetes mellitus family members in the Ethiopian suburban cities Kemisse, and Kombolcha had better knowledge and practice about DM compared to controls. But, the overall awareness about DM occurrence and prevention was relatively low. Thus, DM awareness campaigns should be strongly pursued regardless of family history and educational background to prevent further increase of DM in Ethiopia.

**Electronic supplementary material:**

The online version of this article (10.1186/s13104-017-2871-7) contains supplementary material, which is available to authorized users.

## Background

Diabetes mellitus (DM) consists of a group of common non-communicable diseases, affecting the health of a significant proportion of the population throughout the world. The most common type of DM is type 1 diabetes (T1DM) in which insulin is lacking as a result of failure of the pancreas. Type 2 diabetes (T2DM) is due to the limited ability of the body to respond to the action of insulin. Both types of DM have a complex etiology, and can be caused by mutations in multiple genes, often accompanied by environmental factors [1].

Although DM was once considered as a rare disease in sub-Saharan Africa, over 12 million people of the continent were estimated to have the disease. In the year 2010, about 330,000 people were estimated to die from diabetes-related conditions [2]. It is predicted that sub-Saharan Africa will acquire the highest number of people with DM of any region in the world, reaching up to 23.9 million by 2030 [3]. Ethiopia is located at the horn of Africa, with a total population of over 90 million, most of them living outside of big cities. Although nationwide surveillance assessing the prevalence of DM is lacking, the estimated prevalence in 2012 was 3.32% [3]. In 2013 the prevalence among HIV/AIDS patients taking HAART reached 8% [4]. A study conducted in Jimma, south West Ethiopia, reported that the prevalence of Impaired Glucose Tolerance (IGT) was about 15% [5], suggesting that DM prevalence could be higher than the national estimate of 3.32%, and so could be the associated morbidity and mortality.

The few studies conducted at different places of Ethiopia indicated that DM is becoming a public health problem; however, surveillance targeting on DM prevalence and associated complications is limited. The World Health Organization (WHO) and the American Diabetes Association (ADA) indicated that family history is a main risk factor for development of DM [6, 7]. Inheritance of T1DM may reach up to 30% [8]. In addition, having a first-degree relative with DM is considered an important risk factor to develop T2DM, due to inheritance of genetic risk factors and/or a similar life style pattern among family members [9]. Environmental factors such as over-nutrition and obesity, and life style changes due to increased urbanization and hygiene may also add to the risk of developing T2DM in Ethiopia. Thus, to prevent and control the occurrence of DM in the country, implementation of strategies, such as health education and increased awareness about DM, for DM patients’ family members is essential. This study assessed the knowledge about DM, as well as the practice to prevent DM, of family members of diagnosed DM patients in two suburban cities in Ethiopia. Findings from the study may help to design appropriate intervention strategies.

## Methods

### Study area and population

**Fig. 1:**
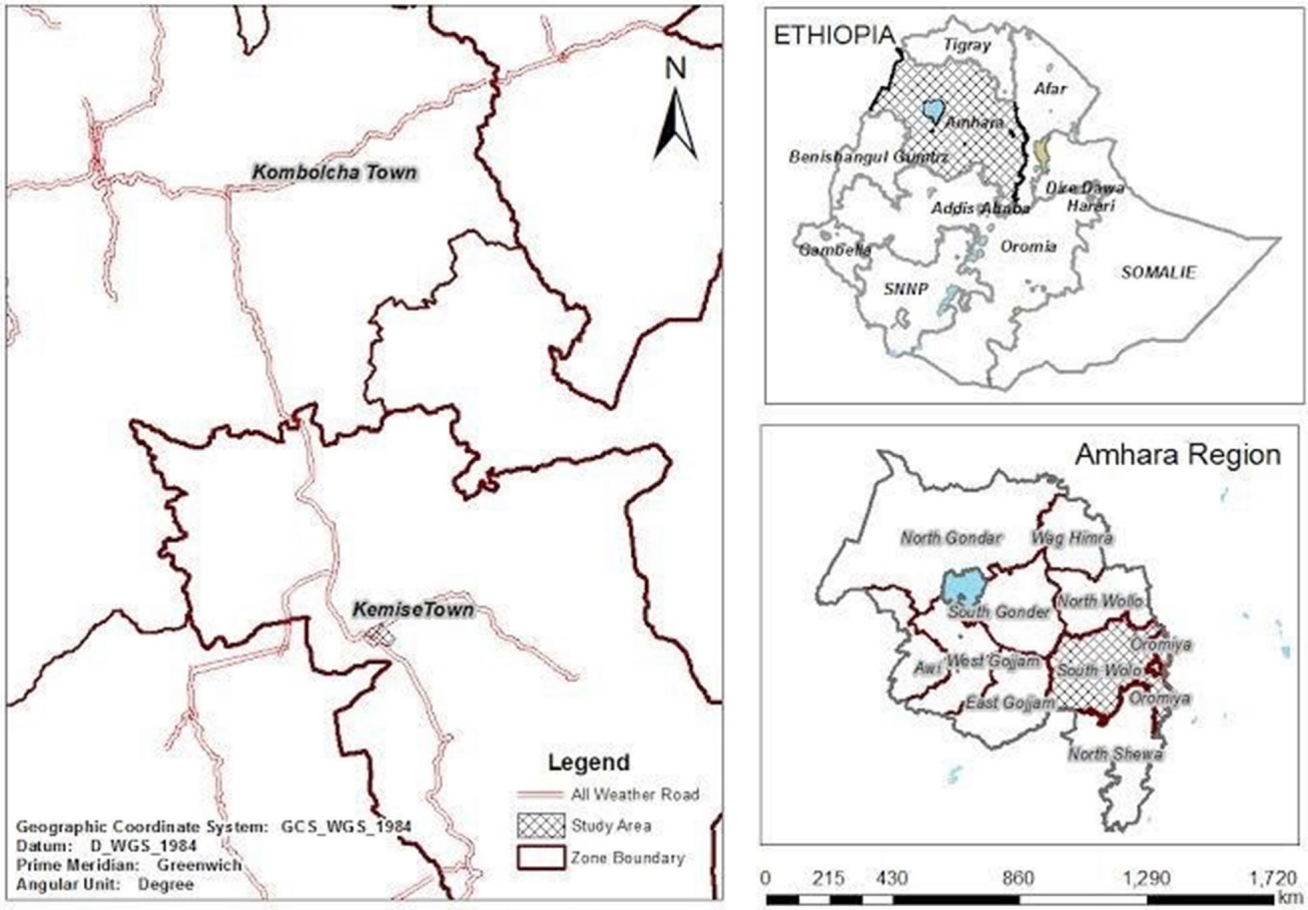
map of the study site (source: central statistics agency of Ethiopia)

This was a cross sectional study, conducted in two geographically close areas of north-east Ethiopia, namely, Kemisse and Kombolcha, respectively (Fig. 1). In the year 2014, the population size of the two study areas were 28,779 and 116,682, respectively [10]. These two study areas had fairly comparable socio-demographic characteristics. Kemisse, is a special woreda of Oromia in the Administrative Zone of Amhara Regional State. The city is located at a distance of 325 km northeast of Addis Ababa, with an altitude of 1450 m above sea level (a.s.l) in the Borkena river basin. Trade, agriculture and livestock productions are the main source of income in the area. Irrigation is practiced for vegetables and *khat* cultivations. Kombolcha, is found in the Amhara Regional State, and located 376 km northeast of Addis Ababa, and 50 km far north of Kemisse. Trade, factory work, and agriculture are the main source of income in the area. [11].

Based on our eligibility criteria, individuals who had primary or secondary degree DM patient family members plus at least one of the WHO categories for increased risk of developing T2DM [6], such as obesity, hypertension, adult age, previously identified impaired fasting glucose or impaired glucose tolerance, reduced physical activity, history of gestational DM or delivery of babies > 4.5 kg, were considered as cases. Controls (basically individuals who do not have DM family members) were recruited as study participants from the two study sites. Those individuals who had been diagnosed of DM were not eligible for the study. Four health extension workers (two from each study site), were trained how to collected data. The health extension workers were given an assignment of going house to house, to identify eligible participants, and invite those consenting for interview.

Information about awareness of DM were collected together with socio-demographic data using interviewer administered questionnaire. Socio-demographic data included information about sex, age, marital status, ethnicity, religion, residence, educational status, and occupation. Twenty questions were developed to assess awareness about the occurrence and prevention practices of DM. Ten of the questions were about knowledge of DM, such as what DM is, its symptoms, risk factors, complications, and source of information about DM In addition, ten “practice” questions like what the study participants do to prevent development of DM, including intake of type of food, regular physical exercise habits, smoking and drinking behaviors, regular checkup of blood sugar, and blood pressure were included to assess practice.

The knowledge and practice categories were defined based on the score attained by each study participant. Each correct knowledge and practice answer had one point. A total score of at least five points (for each knowledge and practice questions separately), was rated as good, and if the cumulative score was below five it was rated as poor.

### Data analysis

Data were entered, cleaned and analyzed using SPSS version 20 statistical software. Descriptive statistics, including frequencies, Chi squares, independent mean tests, and logistic regression were employed for the data analysis. Those variables with P < 0.05 in the bivariate model were included in the multivariable model. These were address, sex, age, educational status, and DM family history. Statistical significant differences among comparable variables were declared when P value was less than 0.05. Results were summarized in tables and figures.

## Results

### Socio-demographic characteristics of study participants

The study aimed to recruit 400 participants (200 from each site). Of them, 171 study participants from Kemisse, and 176 study participants from Kombolcha fulfilled the eligibility criteria and consented for interview.

Of the total 347 study participants considered for awareness study 45.5% (n = 158) had primary or secondary degree DM family member while the remaining 54.5% (n = 189) had no family member with history of DM. The majority (60.8% in DM family members and 73.0% in controls) were males. Mean age of those who had DM family members was less than that of the controls (30.06 vs. 37.38 years, respectively). The majority (51.9%) of the DM family member groups were single while the majority of controls (58.2%) were married. In both study groups, the majority were Junior and high school students (Grade 7–12). Most of the study participants were involved in private business. The overall socio-demographic features of all study participants are summarized in Table 1.

**Table 1:** Associations between having DM family member and socio-demographic characteristic of study participants

Variable	Category	Have DM family member		P value*
		Yes (%)	No (%)	
Sex	Male	96 (60.8)	138 (73.0)	0.015
	Female	62 (39.2)	51 (27.0)	
Age (years)	18–25	65 (41.1)	40 (21.2)	0.001
	26–35	56 (35.4)	68 (36.0)	
	36–45	17 (10.8)	34 (18.0)	
	46–55	14 (8.9)	1 (9.5)	
	≥ 56	6 (3.8)	29 (15.3)	
Marital status	Single	82 (51.9)	53 (28.0)	0.001
	Married	66 (41.8)	110 (58.2)	
	Divorced/widow	10 (6.3)	26 (13.8)	
Educational status	Illiterate	13 (8.4)	30 (16.2)	0.001
	Grades 1–6	17 (11.0)	46 (24.9)	
	Grades 7–12	94 (60.6)	82 (44.3)	
	Higher education	31 (20.0)	27 (14.6)	
Job	Student	39 (24.7)	10 (5.3)	0.001
	Governmental	22 (13.9)	20 (10.6)	
	Private	46 (29.1)	82 (43.4)	
	House wife	13 (8.2)	28 (14.8)	
	Unemployed/other	38 (24.1)	49 (25.9)	
Address	Kemisse	82 (51.9)	89 (47.1)	0.372
	Kombolcha	76 (48.1)	100 (52.9)	

* P value was calculated using Chi squared test

### Knowledge and practice about diabetes mellitus

There was significant difference in knowledge and practice related to DM between those having DM family members and control groups (P < 0.001) (Table 2). The majority of those having DM family members have good knowledge (78.3%) as well as practice (67.3%) compared to those who did not have DM family member (54.5 and 36.3% for knowledge and practice, respectively).

**Table 2:** Knowledge and practice about DM occurrence and preventions among DM family members

Variable	Category	Have DM family member		P value*
		Yes (%)	No (%)	
Knowledge	Good	123 (78.3)	102 (54.5)	0.001
	Poor	34 (21.7)	85 (45.5)	
Practice	Good	105 (67.3)	66 (36.3)	0.001
	Poor	51 (32.7)	116 (63.7)	

* P value was calculated using Chi squared test

### Knowledge and practice of study participants regarding DM and associated background characteristics

Association of background characteristics with good level of knowledge and practice about DM is summarized in Table 3. The likelihood of having good level of knowledge among individuals who had DM family members was 2.94 times (AOR = 2.94 95% CI 1.87–4.86) higher compared to those who did not. Those attaining higher educational levels were 3.41 times (AOR = 3.41, 95% CI 1.31–8.91) more likely to have good level of knowledge as compared to those who were unable to read and write.

**Table 3:** Factors associated with having good level knowledge about DM and positive practice towards DM of study participants in Kemisse and Kombolcha, Northeast Ethiopia, 2014

Variables	Good knowledge (%)	COR with (95% CI)	AOR with (95% CI)	Good practice (%)	COR with (95% CI)	AOR with (95% CI)
Address						
Kemisse	106 (46.7)	1	1	95 (55.2)	1	1
Kombolcha	121 (53.3)	0.75 (0.48–1.18)	1.11 (0.61–2.03)	77 (44.8)	1.77 (1.15–2.72)*	2.33 (1.31–4.12)*
Sex						
Male	159 (70.0)	0.73 (0.46–1.16)	0.81 (0.45–1.48)	109 (63.4)	1.53 (0.97–2.43)	1.11(0.63–1.98)
Female	68 (30.0)	1	1	63 (36.6)	1	1
Age						
18–25	74 (32.6)	1	1	56 (32.6)	1	1
26–35	84 (37.0)	1.15 (0.65–2.02)	0.92 (0.49–1.71)	64 (37.2)	0.47 (0.21–1.06)	0.86 (0.47–1.56)
36–45	35 (15.4)	1.19 (0.58–2.46)	0.78 (0.35–1.73)	23 (13.4)	0.52 (0.23–1.15)	1.04 (0.49–2.20)
46–55	19 (8.4)	1.69 (0.74–3.84)	1.12 (0.44–2.89)	17 (9.9)	0.72 (0.29–1.77)	0.64 (0.25–1.65)
≥ 56	15 (6.6)	3.29 (1.49–7.27)*	1.33 (0.51–3.42)	12 (7.0)	0.47 (0.17–1.28)	1.36 (0.51–3.60)
Educational status						
Illiterate	16 (7.2)	4.48 (1.94–10.42)**	3.41 (1.31–8.91)*	15 (8.9)	2.15 (0.95–4.87)	2.22 (0.84–5.89)
Grade 1–6	36 (16.1)	1.85 (0.86–3.99)	1.27 (0.55–2.98)	28 (16.7)	1.32 (0.64–2.74)	1.04 (0.45–2.41)
Grade 7–12	130 (58.3)	0.91 (0.47–1.77)	0.85 (0.42–1.72)	94 (56.0)	1.03 (0.56–1.87)	0.89 (0.46–1.72)
Higher education	41 (18.4)	1	1	31 (18.5)	1	1
DM family history						
Yes	123 (54.7)	1	1	105 (61.4)	1	1
No	102 (45.3)	3.02 (1.87–4.86)**	2.94 (1.72–5.02)**	66 (38.6)	3.62 (2.31–5.68)**	3.38 (2.05–5.58)**

1: reference group

Multivariate logistic regression adjusted for address, sex, age, educational status, and DM family history

Statistically significant (* p ≤ 0.05, ** p ≤ 0.01)

The likelihood of having good level of positive practice among individuals who were having DM family members were 3.38 times (AOR = 3.38% CI 2.05–5.59) higher as compared to those who did not. Study participants who were living in Kombolcha were 2.33 times (AOR = 2.33 95% CI 1.31–4.12) more likely to have good level of practice, as compared to individuals who were living in Kemisse.

## Discussion

The study reported herein aimed to assess awareness and practices of DM patients’ family members about DM occurrence and preventions, in two sub-urban cities of Ethiopia. In summary having DM family member and higher level educational status were significantly associated with good knowledge and practice regarding DM occurrence and prevention.

Unlike most knowledge, Attitude, and Practice (KAP) studies which describe about DM prevention and control in DM patients in specific areas [12–14], this paper in particular focused on knowledge and practice of DM patients’ family members. Thus, due to the very limited number of similar studies conducted on DM high risk group about DM awareness in the country, we compared our findings mostly with findings of KAP studies done on DM patients.

DM family members are expected to have more chance to be in contact with DM patients at least within their families, and so assumed to have better awareness about DM occurrence and prevention. This study also indicated that 78.3% DM patients’ family members and 54.5% of controls had good knowledge. At the same time, there was more than two fold difference on awareness of good practices on prevention and control of DM between DM patients’ family members and the control groups, 67.3 vs. 36.3%, respectively. Similar findings were seen in a study by Robert et al., which indicated African Americans with a family history of DM were more aware about DM risk factors than those without a family history of the disease [15]. On the other hand, our finding was not in line with a study from South Africa [13]. The difference between our study and that of S. African may result from differences in the sample size (32 study participants in S. Africa versus 347 in our study) and as well on specific objectives and selection of study participants (DM general awareness and treatment approaches in S. Africa, versus a general knowledge and diverse population in our case).

Education is one of the key factors in prevention and control of diseases. In our study, those study participants who were in high school, and those who joined higher education had relatively more awareness about DM occurrences and preventions as compared to those illiterate study participants. This finding has similarity with study conducted in S. Africa, Kenya, and Ethiopia [12, 14, 16]. The association of DM knowledge with academic status may reflect that study participants with better education may have better chance to read information regarding about DM. Such individuals also have more possibilities to communicate with appropriate health personnel and know more about the disease [9].

In general, our study indicated that the overall knowledge and practice of the study participants about DM occurrence and prevention actions was low. Similar finding was seen in a study conducted in Ethiopia [16], Kenya [14] and S. Africa [12]. This may reflect the little attention given to health education for the control of DM in the country since most emphasis in private and public health facilities is given for communicable diseases, such as *Mycobacterium tuberculosis*, Malaria and HIV/AIDS, than the non-communicable ones [17] including DM. There is a civic society associated with DM, but has scarcity of resources to reach most part of the country, and as well as lack of clear guidelines regarding Diabetes mellitus [17, 18].

### Strength and limitations of the study

This study was undertaken among individuals who have DM family members (primary or secondary), and this was not as such common approach regarding DM awareness researches. Moreover, the research being carried out in suburban cities community members with different backgrounds may be considered as a strong point. On the other hand, the study being cross sectional, and due to getting individuals who has/had DM family member in such suburban cities being difficult, and thus included limited number of study participants, limits the generalization of its outcomes.

## Conclusions

The current study demonstrated that individuals with DM family members had better knowledge and practice about occurrence and prevention of DM. Nevertheless, the overall awareness about DM occurrence and prevention by those study participants with DM family members were still unsatisfactory. Therefore, campaign on prevention and control through increasing awareness about DM should be strongly pursued regardless of family history and educational backgrounds.

## Additional file

**Additional file 1.** DM knowledge questions and DM prevention practice questions.

## Abbreviations

a.s.l: above sea level
ADA: American Diabetes Association
AOR: adjusted odd ratio
CI: confidence interval
CO: crude odd ratio
DM: diabetes mellitus
HAART: highly active anti-retroviral therapy
HIV/AIDS: human immunodeficiency virus acquire immunodeficiency syndrome
IGT: impaired glucose tolerance
KAP: knowledge, attitude, and practice
T1DM: type 1 diabetes
T2DM: type 2 diabetes
WHO: World Health Organization
