# Supplementary material for: Knowledge and practice on prevention of diabetes mellitus among Diabetes mellitus family members, in suburban cities in Ethiopia
Source: BMC Res Notes. 2017 Nov 2;10:551. doi: 10.1186/s13104-017-2871-7 (PMC5669018; doi:10.1186/s13104-017-2871-7)
Supplement: Supplementary file 1 — Additional file 1. DM knowledge questions and DM prevention practice questions. [file 13104_2017_2871_MOESM1_ESM.docx]

**DM Knowledge questions**

| **Sr. no.** | **DM knowledge questions** | **Answer options** | **Remark** |
| --- | --- | --- | --- |
| **1** | What is DM? | Raised blood sugar?  I don’t know |  |
| **2** | Do you know types of DM? | T1DM,  T2DM,  GDM,  I don’t know |  |
| **3** | How do an individual acquire DM/risk factors? | Family history?  Eating too much sugar?  Drinking alcohol?  I do not know |  |
| **4** | What are symptoms of DM? | Hunger?  Trusty? Lose weight?  I don’t know |  |
| **5** | Source of information about DM? | Mass media?  Health institution health education?  Family?  Other sources |  |
| **6** | What are complications of DM? | Blindness?  Gangrene?  Stroke?  Any other |  |
| **7** | Is DM contagious disease? | Yes  No |  |
| **8** | Is DM acquired from evil spirit? | Yes  No |  |
| **9** | Is DM curable disease? | Yes,  No |  |
| **10** | Does DM attack all age groups | Yes  No |  |

**DM prevention practice questions**

| **Sr. no.** | **DM prevention questions** | **Answer options** | **Remark** |
| --- | --- | --- | --- |
| **1** | Do you have planned/programmed food consumptions habits? | -Yes, always  -Seldom  -Not at all, and consumed what I got |  |
| **2** | What is your fruit and vegetables eating habits? | -Regularly  -Sometimes  -Not at all |  |
| **3** | What is your meat and fatty food eating habits? | -Regularly  -Sometimes  -Not at all |  |
| **4** | Do you do physical exercises regularly | -everyday?  - Every other day?  -Once in a week?  -Not at all |  |
| **5** | Do you have Smoking habit? | - more than 10 cigarettes per day,  - occasionally smoking  - not smoker |  |
| **6** | What is your alcohol consumption behaviors? | - more than 5 bottles of beer per day,  -just 1-2 bottles beer per day,  - not drunk at all |  |
| **7** | Do you do medical checkup regularly? | -every month,  - every 6 month  - not at all |  |
| **8** | Are you chewing “*Khat*”? | -everyday?  - Every other day?  -Once in a week?  -Not at all |  |
| **9** | Do you follow health educations from different Medias/sources? | -Yes  -No |  |
| **10** | Do you share what you know about DM with others? | -Yes  -No |  |

-
